# Oleic Acid Binding and Exchange Dynamics on NaYF_4_ Nanocrystals

**DOI:** 10.1021/acs.jpclett.6c02516

**Published:** 2026-09-03

**Authors:** Ayla J.H. Dekker, Marco Kikkert, Francesca Lavore, Léon Witteman, Markus Weingarth, Pieter C.A. Bruijnincx, Freddy T. Rabouw

**Affiliations:** † Soft Condensed Matter & Biophysics, Debye Institute for Nanomaterials Science, 8125 Utrecht University, Utrecht 3584 CC, The Netherlands; ‡ Organic Chemistry & Catalysis, Institute for Sustainable and Circular Chemistry, 8125 Utrecht University, Utrecht 3584 CG, The Netherlands; § NMR Spectroscopy, Bijvoet Centre for Biomolecular Research, 8125 Utrecht University, Utrecht 3584 CH, The Netherlands

## Abstract

Lanthanide-doped NaYF_4_ nanocrystals (NCs) are widely used for their exceptional photoluminescence properties. Their performance and processability are critically dependent on surface-bound ligands such as oleic acid. While these ligands stabilize NCs during synthesis and in nonpolar solvents, applications often require post-synthetic ligand exchange. Here, we investigate the protonation state, surface ion binding, and exchange dynamics of oleic acid on NaYF_4_ NCs using a variety of analysis techniques, including infrared spectroscopy (IR), nuclear magnetic resonance spectroscopy (NMR), and thermogravimetric analysis (TGA). Our methods distinguish bound and free ligand populations, quantify surface coverage, and detail how common purification steps alter ligand density. These findings offer key insights into NC surface chemistry and provide a foundation for rational NC purification and ligand-exchange strategies, with implications for optimization of colloidal stability and functionalization.

## Graphical abstract

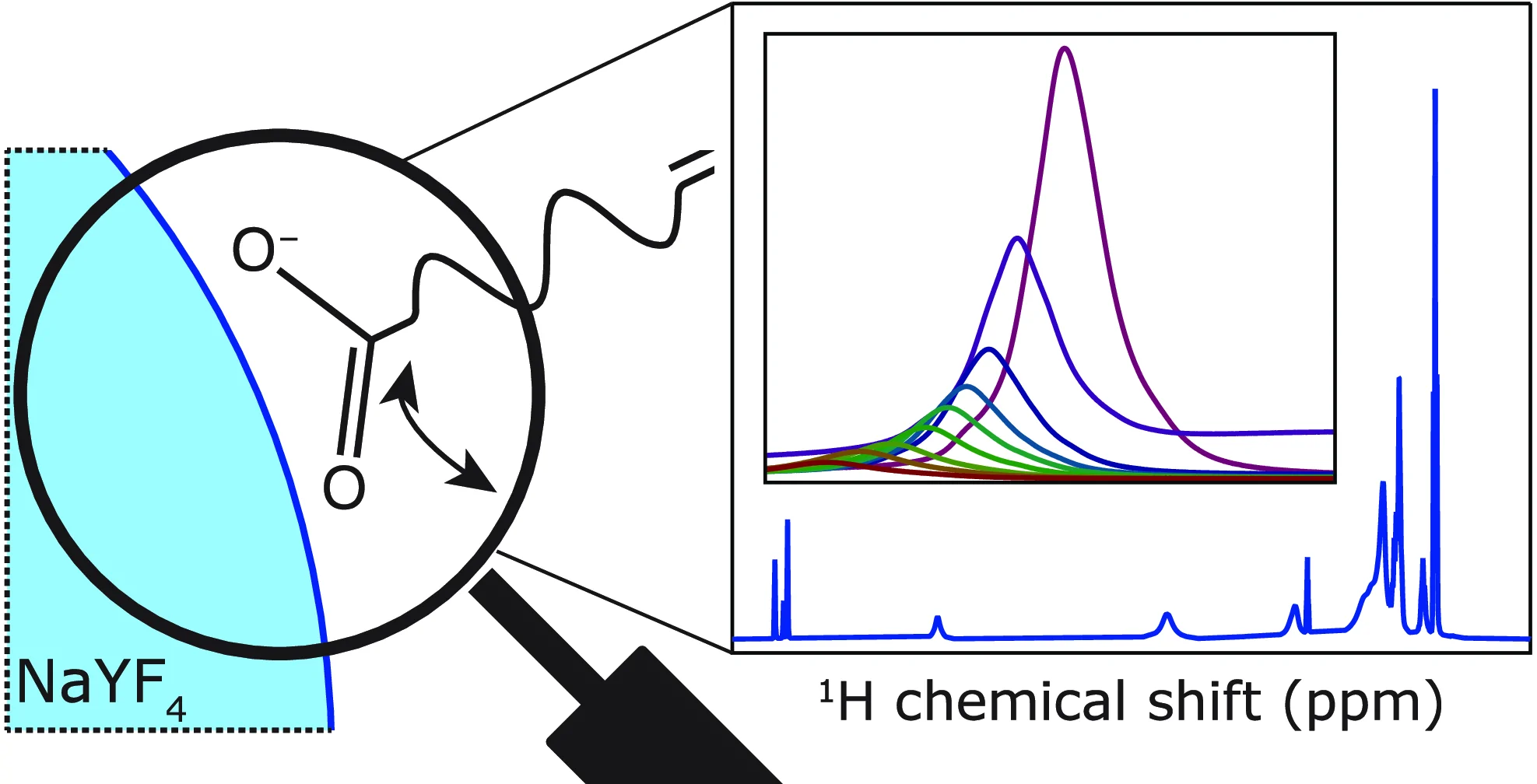

Lanthanide-doped nanocrystals (NCs) offer highly tunable photoluminescence properties, stemming from the diverse electronic transitions of trivalent lanthanide ions.^1–3^ By carefully selecting and combining dopants, the absorption and emission of light can be tuned across a broad spectral range from the ultraviolet to the infrared. This makes lanthanide-doped NCs attractive for applications in bioimaging,^4,5^ drug delivery,^6,7^ sensing,^8,9^ and (nano)­thermometry.^10,11^ Among the many possible host lattices, sodium yttrium fluoride (NaYF_4_) stands out for its positive effect on luminescence quantum yields, while its robust (photo)­chemical stability and high crystallinity promote long-term luminescence performance.^12,13^

The colloidal stability of (doped) NaYF_4_ NCs in dispersion depends critically on their surface chemistry. As-synthesized NCs are typically capped with long-chain organic ligands, most commonly oleic acid. These ligands not only stabilize the nanocrystals during high-temperature synthesis, yielding highly crystalline and monodisperse particles, but also impart dispersibility in nonpolar organic solvents.

Post-synthetic ligand exchange strategies are commonly employed to render the NC surface hydrophilic or to introduce functional moieties such as targeting groups or dyes.^14–19^ In such systems, the nature of the ligand–surface interaction is crucial: strong and stable ligand binding is essential to ensure long-term colloidal stability and to enable efficient energy transfer between surface-bound dyes and the lanthanide ions in the core. Yet, despite the critical role of surface chemistry in determining both fundamental and application-driven properties, some aspects such as binding site and exchange behaviour of ligands on NaYF_4_ NCs remain poorly understood. Moreover, while commonly used purification processes such as washing have been shown to reduce ligand density and surface coverage,^20^ quantitative understanding is lacking, making counter-measures such as ligand addition to the NC dispersion usually guesswork—to the detriment of sample purity.

In this work, we present a comprehensive investigation of the protonation state, surface ion preference, and exchange dynamics of oleic acid and related probe molecules on NaYF_4_ nanocrystals. To this end, we develop and apply nuclear magnetic resonance spectroscopy (NMR) methodologies, including solution and solid-state techniques, assisted by infrared spectroscopy (IR) and thermogravimetric analysis (TGA) to characterize ligand binding at the molecular level. Our approach allows us to distinguish bound from free species, map surface coverage, and probe exchange kinetics. Through this, we not only gain new insights into the surface chemistry of NaYF_4_ NCs, but also provide a framework for the rational design of ligand exchange strategies and surface modifications. The techniques established here are widely available and broadly applicable to other colloidal nanomaterials, although care must be taken to ensure that the nanocrystals do not contain substantially different types of binding sites. Our results contribute to a deeper understanding of nanocrystal–ligand interactions, which are central to the development of functional (hybrid) nanosystems.

## Ligand Binding

We study undoped NaYF_4_ NCs capped with oleic acid (OA), synthesized using the thermal decomposition method.^21^ In most studies and applications, these NCs would be doped with lanthanide ions by randomly replacing Y^3+^ ions in the crystal lattice, providing the desired optical properties. However, the paramagnetic nature of optically active trivalent lanthanide ions can substantially broaden and shift NMR signals. As this would hamper the type of analysis we aim to perform, our NCs contain no paramagnetic lanthanide ions. Our results on undoped NCs are nevertheless representative of trivalent-cationic-lanthanide-doped NCs, as the chemical properties among the rare earths are very similar.^22^

Figure 1a shows the NMR spectrum of freely dissolved OA (red, top) and dispersed oleic-acid-capped NaYF_4_ NCs (blue, bottom), both in deuterated toluene, zoomed in on the alkene (5.35–5.75 ppm) and carboxylic acid (9–13.5 ppm) regions. The alkene resonances of bound OA broaden and shift downfield compared to freely dissolved OA, such that proton multiplicity is obscured. Both broadening and peak shift are consistent with OA binding on other NCs, such as PbSe, PbS, CdSe, and HfO_2_ NCs.^23–28^ Another notable difference between the spectra is the absence of a resonance between 13 and 9 ppm for NCs, while the free OA shows a clear carboxylic proton signal. Likely, this proton is lost upon binding, or its signal is broadened to an extent that it no longer exceeds the noise.

**Figure 1:**
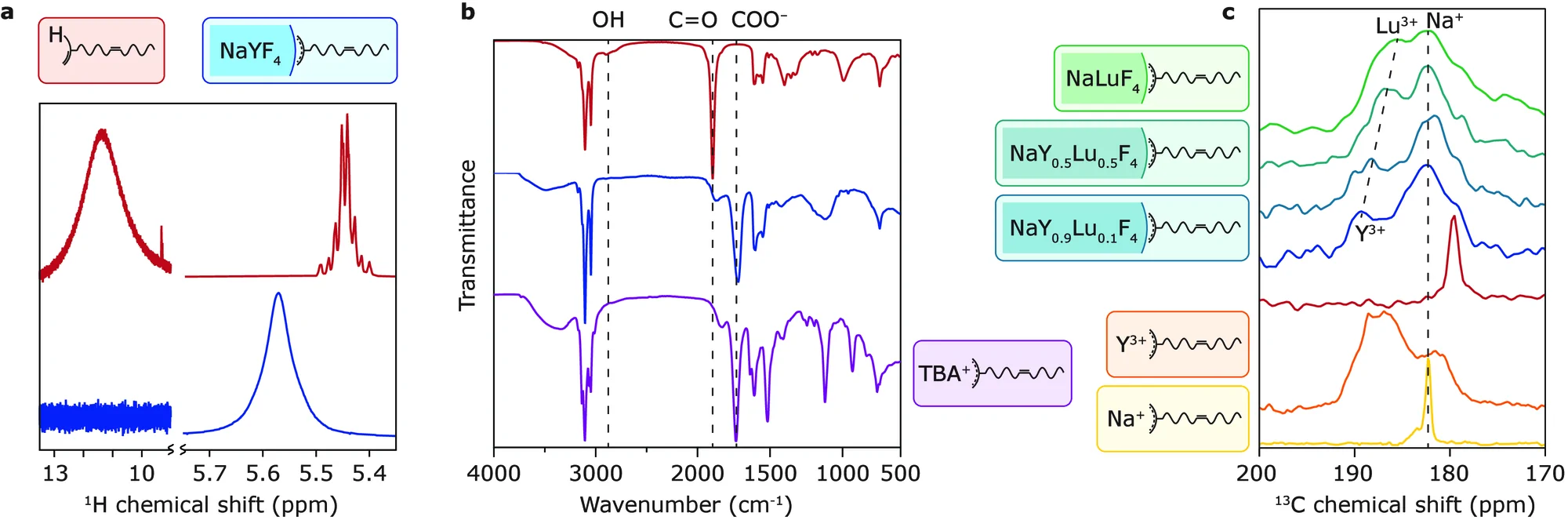
(a) ^1^H NMR spectra of oleic acid (top, red) and oleic acid-capped NaYF_4_ nanocrystals (bottom, blue) in deuterated toluene with the measured species shown schematically above the spectra in corresponding colours. The spectra are zoomed in on the acidic (13.5–9 ppm) and alkene (5.75–5.35 ppm) proton regions. (b) IR spectra of oleic acid (red), oleic acid-capped NaYF_4_ nanocrystals (blue), and TBA oleate (purple), all pure/dry without any solvent. Dashed lines indicate characteristic vibrations for functional groups indicated above. (c) Zoom into the carbonyl region of ^13^C NMR spectra. In red: the solution NMR spectrum of oleic acid in deuterated toluene. The other spectra show solid-state NMR of dried sodium oleate (yellow) and dried yttrium oleate (orange). The blue-to-green gradient shows dried oleate-capped NCs, where the composition is varied from NaYF_4_ (blue) to NaLuF_4_ (green) with NaY_0.9_Lu_0.1_F_4_ and NaY_0.5_Lu_0.5_F_4_ in-between.

The binding state of OA to NaYF_4_ NCs was further established with the use of infrared (IR) spectroscopy. In Figure 1b, we compare the IR absorption of bound OA, free OA, and tetrabutylammonium oleate (TBAOl). The latter is an ionic liquid, consisting of oleate and the bulky cation TBA. Notably, the C=O stretch (1710 cm^–1^) and broad carboxylic OH vibrations (2400 – 2800 cm^–1^) are present in the OA spectrum, while absent in the TBAOl and NC spectra.^29,30^ For the antisymmetric COO^–^ stretch absorption (1570 cm^–1^) the opposite is true.^30,31^ Thus, IR absorption of the NC ligands resembles TBAOl more closely than OA, indicating that OA binds to NCs in its deprotonated oleate form.

As a negatively charged ion, oleate is likely bound to a positive surface ion. In NaYF_4_ these are Na^+^ or Y^3+^. To understand this binding in more detail, Figure 1c shows ^13^C NMR measurements of the carboxyl carbon. Being closest to the NC surface, the carboxyl carbon is most sensitive to ligand binding mode and site. Liquid OA in deuterated toluene (red) shows a single sharp carboxylic resonance. This splits, broadens, and shifts downfield upon deprotonation and binding to NaYF_4_ NCs (blue). When a larger fraction of yttrium is replaced with lutetium (blue-to-green gradient) the leftmost peak shifts slightly upfield, while the right peak remains at the same position. Since the rare-earth composition varies and the sodium content remains constant, it stands to reason that the left, shifting peak corresponds to rare-earth-bound oleate, while the right, constant peak arises from sodium-bound oleate. However, XRD measurements also show that the crystal structure partly shifts from hexagonal to cubic phase when Y^3+^ is replaced by Lu^3+^ (Figure S2), which can also affect the NMR spectrum. Comparing solid-state ^13^C NMR spectra of sodium oleate (yellow) and yttrium oleate (orange) to the NC spectra, we see a single peak for sodium oleate in line with the upfield peak of all NCs. While yttrium oleate also has a small peak approximately at this shift, it has a stronger resonance aligned with the oleate-capped NaYF_4_ peak further downfield. This also indicates that the resonance downfield arises from oleate bound to yttrium, while the resonance upfield is predominantly from sodium-bound oleate. Although firm claims are difficult with the different crystal structures, we tentatively conclude that oleate binds to both sodium and rare-earth ions.

Going forward, bound oleate ligands and oleic acid freely dissolved will both be abbreviated with OA, as they are occasionally referenced as a collective.

## Ligand Exchange and Kinetics

NCs are usually washed after synthesis to remove reactants, by-products and solvent. However, washing also reduces ligand density, often to the detriment of colloidal stability. To study this, we washed a batch of NCs seven times, taking a sample after each wash. Figure 2a shows the steps in a single wash cycle. This also illustrates how ligand density is lost, as free ligands are discarded with the supernatant and bound ligands detach upon subsequent re-dispersion. The relative amounts of free and bound ligands are dictated by the equilibrium between the binding of free OA to a vacant site and the reverse process, with equilibrium constant *K*. Usually *K* refers to the equilibrium in a stable dispersion in a pure solvent, but here we subject the NCs to precipitation in a toluene/ethanol mixture where the equilibration time is approximately 5 min before centrifuging and discarding the supernatant. NC aggregation and altered solvent properties affect the binding–unbinding equilibrium of OA. Therefore, *K* should be regarded as an apparent equilibrium constant for this particular washing process, not an inherent thermodynamic binding constant. The total remaining OA at the end of a wash (stage V) is equal to the amount of *bound* OA before centrifugation (stage II) plus the free OA in the small amount of solvent (d*V*, see Section S1.13 of the SI) trapped within the precipitate upon decanting (stage IV).

**Figure 2:**
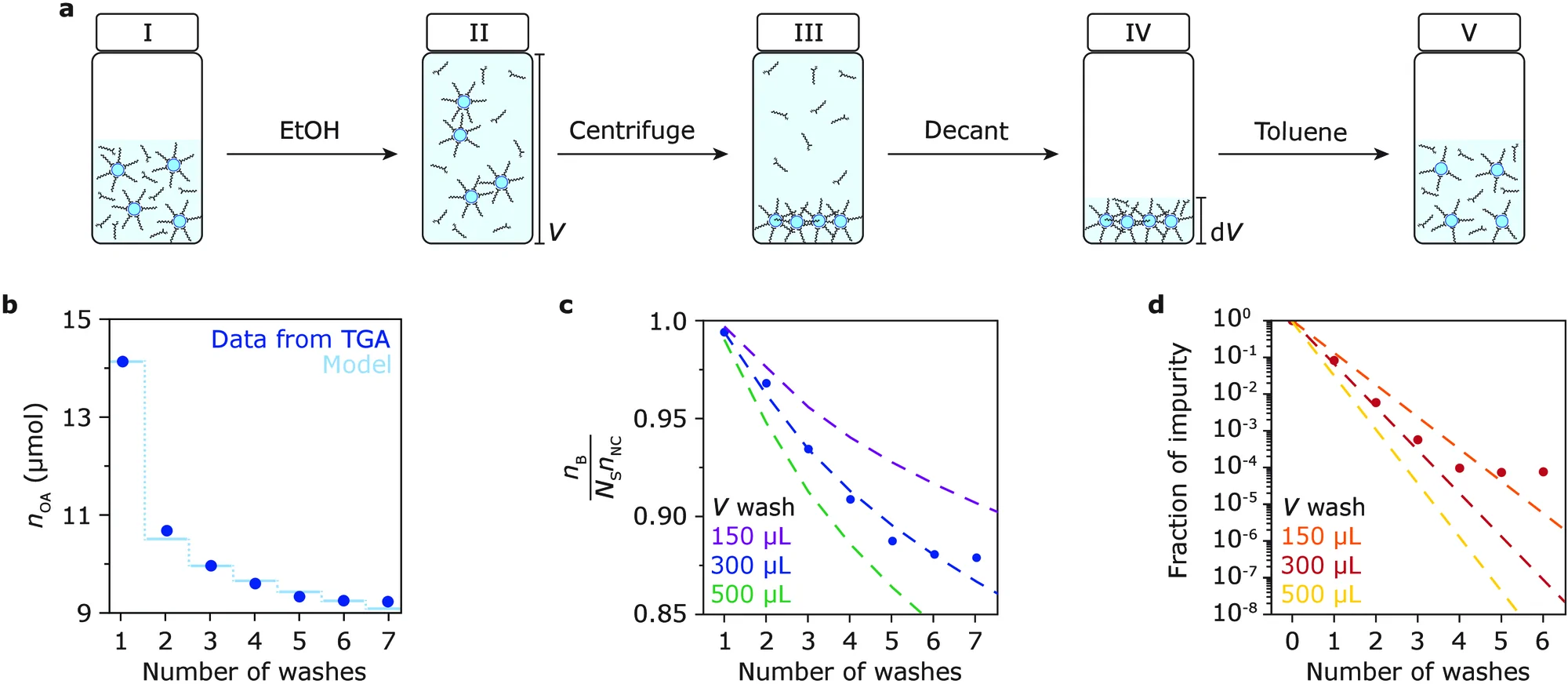
(a) Schematic of the washing procedure. The total amount of OA in stage V is the amount of OA bound in the stage II plus the free OA trapped between the precipitate in stage IV. (b) The amount of OA as a function of the number of washes (dark blue circles) as determined by thermogravimetric analysis. Section S4 of the SI explains details of the method. The light blue step function shows the OA content evolution as modeled. (c) Ligand coverage as a function of number of washes. Dashed lines: calculated by iterating eq 1 using the fit parameters determined in panel b and for different washing volumes *V*. Data points: experimental *n*_OA_ values of panel b where *V* = 300 *μ*L converted to *n*_B_ using eq 1. (d) Fraction of impurity remaining as a function of number of washes. Red circles are data points for an added dye, its concentration is derived from its photoluminescent intensity. Dashed lines show (d*V*/*V*)^*n*^ for different values of *V*.

Figure 2b shows the OA content of the different samples, measured with thermogravimetric analysis. The loss of OA is determined by the binding–unbinding equilibrium just before centrifugation (stage II). The equilibrium constant *K* (unit: M^–1^) relates the amount of bound OA (*n*_B_) and the total OA (*n*_OA_):

1 $$K=\frac{n_{B}V}{\left(n_{\mathrm{OA}}-n_{B}\right)\left(N_{S}n_{\mathrm{NC}}-n_{B}\right)}$$

Here, *N*_S_ is the number of OA binding sites per NC and *n*_NC_ the total amount of NCs (unit: mol), and *V* is the volume in stage II. With the OA content after the first wash as input, the OA amount after all subsequent washes can be calculated, with *K* and *N*_S_ as fit parameters, and accounting for the bit of free OA trapped in d*V* (for details see Section S6 of the SI). The data is best described with values of *K* = 1.3 × 10^4^ M^–1^ and *N*_S_ = 327, yielding the step function in Figure 2b. Here, we find a single, average value for *K*. By describing the ligand binding with a single *K* value, we further neglect any potential variations in binding strength due to, for example, different crystal faceting. This simplified model fits the data well.

To our knowledge, no literature data on the binding–unbinding equilibrium of OA on NaYF_4_ NCs is available. Our fitted value of *K* (∼10^4^) is lower than for the binding–unbinding of Cd-oleate on CdS quantum dots in benzene and cyclohexane (10^5^–10^6^ M^–1^)^32^ but higher than for Cd-oleate on CdS (∼ 10_^2^_ M^–1^) in THF.^33^ Our fitted value of *N*_S_ = 327 corresponds to 2.7 sites per nm^2^, as the NCs are approximately spherical with an average radius of 3.1 nm (Figure S1a). A study by Tong *et al.* reports an OA density of 8.9 nm^–2^ for NaYF_4_ NCs washed with hexanes and ethanol.^20^ While seeming incongruent, it should be noted that the authors state that 8.9 nm^–2^ exceeds the theoretical limit of OA binding on a surface and postulate that a significant part of the OA is trapped in the shell of hydrocarbon chains of the bound ligands, thus not occupying binding sites. Encouragingly, they report an OA density of 2.6 nm^–2^ after washing with THF and ethanol. This value being essentially equal to the 2.7 binding sites nm^–2^ perhaps indicates that redispersing in THF does remove the physically trapped OA molecules, leaving only bound ligands. 2.7 nm^–2^ also matches well with values reported for OA surface density on semiconductor NCs: 1.4, 1.8, and 4.6 nm^–2^ on CdSe,^28,34,35^ 3.0 nm^–2^ on PbS,^26^ and 4.2 nm^–2^ on PbSe.^24^

Understanding the binding–unbinding equilibrium of OA can help in the design of efficient washing strategies. Figure 2c shows the fraction of binding sites filled as a function of number of washes for various washing volumes. Here, we find *n*_B_ for each washing step by iterating eq 1, starting with the *n*_OA_ after the first wash measured with thermogravimetric analysis as input. Dividing by the total number of binding sites (*N*_S_ *n*_NC_) gives ligand coverage. By simply changing the value of *V* in eq 1, the effect of a larger (green line) or smaller (purple line) washing volume on ligand density can be easily predicted. We can also re-plot the data in Figure 2b by calculating the *n*_B_ from each data point *n*_OA_ with eq 1, again normalized by number of binding sites. These data points follow the theoretical with the actual volume (300 *μ*L, blue) well.

Simultaneously, we study how effective washing is by getting the concentration of a dye (coumarin 6H) in the sample after each wash from the total emission intensity of photoluminescent measurements (Figure S9). Figure 2d shows the fraction of this dye remaining (*y*) as red circles. Mostly, this scales with the fraction of solvent trapped in stage IV of the washing procedure and number of washes as *y* = (d*V*/*V*)^*n*^, shown as the red dashed line. After approximately 4 washes this line deviates from the experiment, likely due to coordination of a small fraction of the dye to the NCs. Like ligand density, we can calculate the impurity fraction for multiple volumes (yellow and orange dashed lines). Here, we assume d*V* to be the same, even for different *V*, as long as the amount of NCs remains constant. With these plots we can easily see the effect of washing strategy (number and volume) on the ligand density and sample purity. Depending on the requirements for the NC application, one can now strategically design the washing method.

We further studied the binding–unbinding by titrating additional OA to a dispersion of oleate-capped NCs in deuterated toluene. Figure 3a shows the alkene resonance in the ^1^H NMR spectrum during titration. We observe a single resonance peak that shifts from 5.51 ppm (red; no added OA) to 5.40 (purple; 9.1 equiv of OA). This result is qualitatively different from previous studies on semiconductor NCs, where titration yielded two distinct peaks from bound and free OA.^35^ The observation of just a single resonance peak here signals that OA binds and unbinds on time scales faster than the NMR experiment, such that the alkene resonance averages out to a single NMR peak. Specifically, the observation sets a lower limit to the OA exchange rate *k*_exc_:^36^

$$k_{\mathrm{exc}}>\frac{2\pi \left(\nu_{B}-\nu_{F}\right)}{2\sqrt{2}}$$ 2

where *ν*_B_ and *ν*_F_ (unit: Hz) are the resonance frequencies of the alkene protons in bound and free OA.

**Figure 3:**
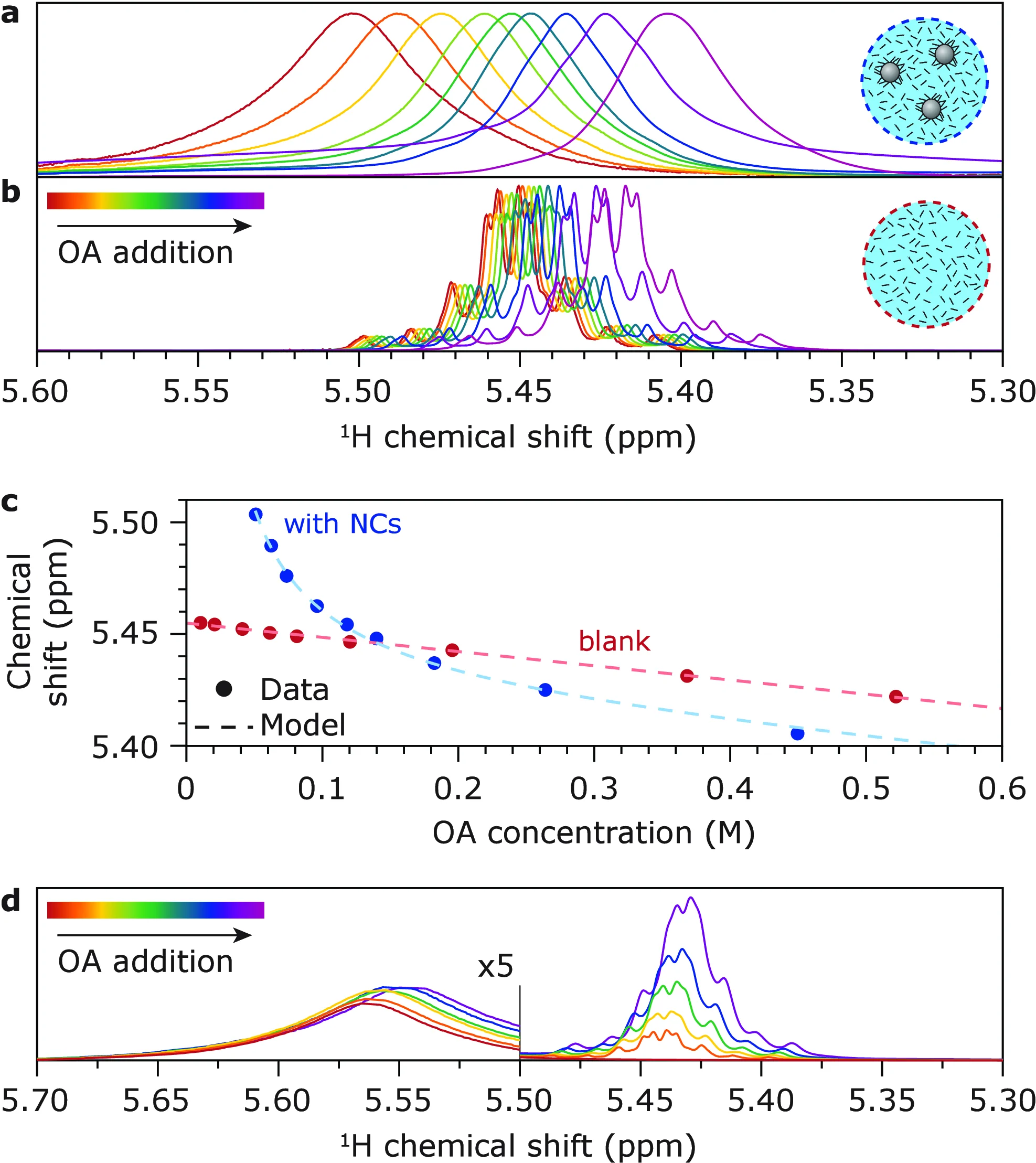
(a) ^1^H NMR spectra zoomed in and normalized on the OA alkene peak. The red curve is from only oleate-capped NCs dispersed in deuterated toluene. Additional OA was added stepwise to this dispersion until 9.1 equiv, shown progressively in various shades of orange, yellow, green, blue, and purple. (b) Same, but the OA was added to deuterated toluene without NCs in dispersion. (c) The peak positions in panels a (blue) and b (red) plotted against total OA concentration (circles), fitted with eqs 4 and 3 (dashed lines), respectively. (d) Same as a, but instead of regular OA, deuterated OA-*d*1 was added where the acidic proton was replaced with a deuterium.

As the separate values of *ν*_B_ and *ν*_F_ are not directly observable in Figure 3a, we performed reference measurements and modeling. Titrating OA in pure deuterated toluene reveals a gradual upfield shift of the alkene resonance (Figure 3b), which likely reflects changes in solvent polarity. We fit the chemical shift as a function of OA concentration in toluene (Figure 3c, red circles) to a linear trend:

3 $$\delta_{\mathrm{OA}}^{\mathrm{pure}}=\delta_{F}+\Delta_{\mathrm{OA}}\frac{n_{\mathrm{OA}}}{V}$$

Here, *δ*_F_ = 5.45 ppm is the chemical shift of free OA in toluene in the limit of low concentration and *Δ*_OA_ = −0.0636 ppm M^–1^ describes how the alkene resonance changes with OA content in the toluene solution. Next, the chemical shifts measured in the titration with NCs (blue data points in Figure 3c) are fitted to

4 $$\delta_{\mathrm{OA}}^{\mathrm{NCs}}=\frac{\delta_{F}\left(n_{\mathrm{OA}}-n_{B}\right)+\delta_{B}n_{B}}{n_{\mathrm{OA}}}+\Delta_{\mathrm{OA}}\frac{n_{\mathrm{OA}}}{V}+\Delta_{\mathrm{NC}}\frac{n_{\mathrm{NC}}}{V}$$

with the chemical shift of bound OA in the limit of low solute concentration *δ*_B_ and the change in OA resonance with NC concentration *Δ*_NC_ as free fit parameters. The first term in eq 4 describes that the fast exchange between free and bound OA lead to a weighted average chemical shift. We find *δ*_B_ = 5.58 ppm and *Δ*_NC_ = −230 ppm M^–1^. We have determined these values solely from the NMR peak position, without relying on the solvent-dependent equilibrium constant *K*.

The fitted values of *δ*_F_ and *δ*_B_, at the 400 MHz frequency of our NMR instrument, set the lower limit of the OA exchange rate at *k*_exc_ > 115 s^–1^. While the exchange rate of strongly-bound oleate on quantum dots is usually reported to be much slower (no exchange on the time scale of an NMR measurement),^24,37^ there have also been reports of more weakly bound OA which have similar exchange rates of >300 s^–1^ (CdSe) and 300–490 s^–1^ (PbS).^35,37^ Low-temperature NMR experiments show that the alkene resonance remains a single exchange-averaged peak at temperatures down to at least −60°C (Figure S10). This indicates that OA exchange on NaYF_4_ is faster than 115 s^–1^ even at low temperatures.

Titrating with deuterated OA-*d*1 (with the acidic proton replaced by deuterium) produces a double-peak alkene resonance in the NMR spectrum (Figure 3d). The bound-OA signal remains constant in intensity, while a second, free-OA signal increases in intensity with added OA-*d*1. Clearly, surface-bound oleate exchanges much more slowly with the deuterated acid, indicating that the proton exchange is an important step in the process, which is significantly slowed down with the heavier deuterium substitution.

Finally, we monitor ligand exchange using NMR spectroscopy, investigating two ligands: undec-10-en-1-ylphosphonic acid (UP) and undec-10-enoic acid (UA). Figure 4 shows the titrations, where an increasing amount of UA (a) or UP (b) is added to oleate-capped NaYF_4_ NCs. The peaks between 5.4 and 5.7 ppm are from the OA alkene hydrogens, the spectra are normalized to these peak heights. The peaks to the right (4.9–5.3 ppm) and left (5.8–6.1 ppm) are due to the titrating ligand’s hydrogens 1 and 2, respectively. Upon addition of the other ligand, the OA peak shifts upfield and narrows. This indicates that the free-to-bound OA ratio increases while the exchange dynamics remain faster than 115 s^–1^. As the titrating ligand concentration increases, their peaks shift as well, consistent with rapid exchange dynamics between bound and an increasing amount of free titrating ligand.

**Figure 4:**
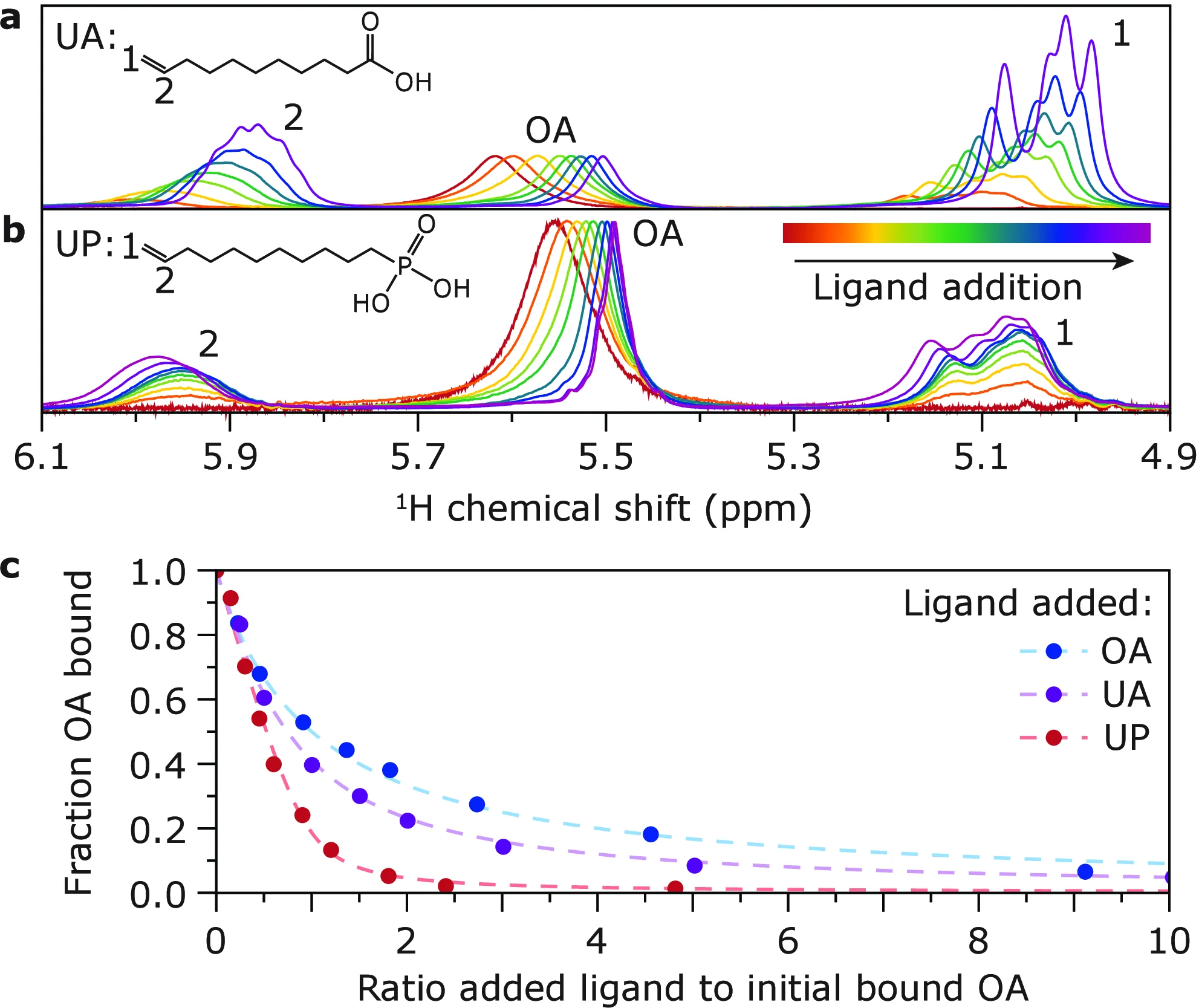
(a) ^1^H NMR spectra zoomed in on the alkene region. The red curve is from only oleate-capped NCs dispersed in deuterated toluene. Undecenoic acid was added stepwise to this dispersion, shown progressively in various shades of orange, yellow, green, blue, and purple. (b) Same, but undecenylphosphonic acid was added. (c) The fraction of OA molecules that are bound to the NC surface as a function of equivalents added ligand with respect to OA already present on the NCs. The circles are data from the titrations in (a), (b), and Figure 3a. Dashed lines are models.

We can quantify OA displacement from the OA alkene peak position. First, we correct for the peak shift caused by increased ligand concentration, assuming that the value of *Δ*_OA_ from Figure 3c describes the influence of OA on chemical shift as well as that of the titrating ligands. As a first step in the analysis, we also assume the OA to be completely bound at the start and completely free at the last point in the titration. We then convert the alkene peak positions to the fraction of OA bound, as a function of the amount of titrating ligand added (Figure 4c). The experimental data follow a chemical-equilibrium model where two ligands compete for the same NC binding sites (Section S7 of the SI). Using this model, we fit ratios of the equilibrium constants for the titrating ligands and OA: *K*_L_/*K*_OA_ with L = UA, UP. Using these ratios, we adjust our initial assumption of zero bound OA at the end of the titration, then redo the conversion of NMR peak position to fraction of OA bound, and fit updated values of *K*_L_/*K*_OA_. This iterative procedure is continued until it converges to stable values of *K*_L_/*K*_OA_.

The results of this procedure and the fits are shown in Figure 4c for titration with OA (blue; reproduced from Figure 3), UA (purple), and UP (red). The titration with OA follows a chemical-equilibrium model with *K*_L_ equal to *K*_OA_ (eq S12), as expected. In the case of UA and UP, we fit *K*_UA_/*K*_OA_ = 2.07 and *K*_UP_/*K*_OA_ = 18.9. This means that the other two ligands bind more strongly than OA. This difference is small between OA and UA, likely caused by the lower solubility of UA in toluene, promoting binding of the polar group to the NC surface or by more steric hindrance for OA making binding less favorable. Similar values of 2.14 and 2.23 are reported for *K*_UA_/*K*_OA_ on PbS NCs.^38^ Interestingly, investigations on similarly sized CdSe also dispersed in toluene-*d*8 yield a ratio of less than one (0.83), showing that UA does not universally bind more strongly than OA.^34^ UP binds much more strongly to the NC surface than either carboxylic acid, since the phosphonic acid is a stronger binding group, consistent with earlier work on quantum dots.^34^

We have used a range of analysis techniques to identify the binding state of OA to NaYF_4_. ^1^H NMR combined with IR spectroscopy determined that OA is bound as the deprotonated oleate. With solid-state ^13^C NMR we distinguished between oleate bound to Y_3+_ and Na^+^ and found that both are present on NaYF_4_. We quantified the binding strength of OA on NaYF_4_ by washing NCs multiple times and tracking the OA concentration. Fitting a chemical equilibrium to the bound OA fraction yielded an equilibrium constant of *K* = 1.3 × 10^4^ M^–1^ and a binding site density of 2.7 nm^–2^. We found that OA exchange on NaYF_4_ is faster than the time scale of the NMR experiment, with a lower limit for the exchange rate of 115 s^–1^. Finally, we monitored ligand exchange, achieving rapid displacement of nearly all OA by titrating undecenoic acid or undecenylphosphonic acid and quantifying the equilibrium constants. Our work demonstrates the significant effects of washing on ligand density, highlighting the need to minimize unnecessary washing, carefully control the (anti)­solvent volume used during washing, and quantify the final ligand coverage when this may influence NC stability. For ligand exchange, the importance of selecting ligands with appropriate binding affinities and concentrations is highlighted. In this way, our results assist in the development of improved NC purification strategies that retain colloidal stability, as well as ligand-exchange strategies for processing and bioconjugation.

Our results can assist in the development of better NC-purification strategies retaining colloidal stability or of ligand-exchange strategies for processing and bioconjugation.

## Supplementary Material



## Notes

Experimental section, transmission electron microscopy and X-ray diffraction of the NCs, full NMR spectra, full washing procedure, thermogravimetric analysis, photoluminescence measurements, variable-temperature NMR, derivation of equations (PDF)
