# Supplementary material for: Oleic Acid Binding and Exchange Dynamics on NaYF4 Nanocrystals
Source: J Phys Chem Lett. 2026 Sep 3;17(37):10640–6. doi: 10.1021/acs.jpclett.6c02516 (PMC13592085; doi:10.1021/acs.jpclett.6c02516)
Supplement: Supplementary file 1 [file jz6c02516_si_001.pdf]

Oleic acid binding and exchange dynamics on $NaYF_4$ nanocrystals

Ayla J.H. Dekker,[†,‡] Marco Kikkert,[†,‡] Francesca Lavore,[¶] Léon Witteman,[‡] Markus Weingarth,[¶] Pieter C.A. Bruijnincx,[‡] and Freddy T. Rabouw[*,†]

†*Soft Condensed Matter & Biophysics, Debye Institute for Nanomaterials Science, Utrecht University, Utrecht 3584 CC, The Netherlands*

‡*Organic Chemistry & Catalysis, Institute for Sustainable and Circular Chemistry, Utrecht University, Utrecht 3584 CG, The Netherlands*

¶*NMR Spectroscopy, Bijvoet Centre for Biomolecular Research, Utrecht University, Utrecht 3584 CH, The Netherlands*

E-mail: f.t.rabouw@uu.nl

S1 Experimental methods

S1.1 Chemicals

The chemicals listed were used without further purification steps unless mentioned otherwise. Sigma Aldrich: Oleic acid (90%, OA), yttrium(III) acetate tetrahydrate (99.99%), lutetium(III) acetate hydrate (99.9%), diethyl ether anhydrous (≥99.0%), triethyl phosphite (98%), deuterium chloride solution 1M in diethyl ether (97.5 atom % D), 1-octadecene (90%), yttrium(III) chloride (99.99%), coumarin 6H (99%). Thermo Fisher Scientific: Tetrabutylammonium hydroxide 1M solution in methanol, p-Tolualdehyde (99%), Ammonium fluoride (98%), n-Hexane (99%), 10-undecenoic acid (99%, UA). VWR Chemicals: Methanol (technical grade), Ethanol (technical grade), Acetone (technical grade), Toluene (technical grade), Isopropanol (technical grade). 11-Bromo-1-undecene (96%) was purchased from Brunschwig Chemie. Dichloromethane (technical grade) was purchased from Biosolve. Pentane (95%) and Hydrochloric acid (30%, technical grade) were purchased from Boom. Sodium Oleate (≥97%) was purchased from TCI. Methanol-d_4 (99.8 atom % D) and Toluene-d_8 (99.5 atom % D) were purchased from Cambridge Isotope Laboratories Inc.. Reactions carried out under N_2 atmosphere were performed with Schlenk techniques.

S1.2 Nanocrystal synthesis

The synthesis of $NaY_xLu_{1-x}F_4$ was carried out based on the method of Homann *et al.*[1] 7.5 mmol rare-earth acetates in the desired $Y(Ac)_3$:$Lu(Ac)_3$ ratio was added with 37.5 mmol sodium oleate, 75 mL ODE and 75 mL OA to a 500 mL round-bottom flask. This was degassed *in vacuo* at 100 °C for one hour, followed by three pump-purge cycles of N_2 and vacuum. 60 mmol NH_4F was added under N_2 flow while maintaining a temperature of 100 °C, followed by three quick pump-purge cycles of N_2 and vacuum (3–5 seconds). The mixture was heated to 310 °C (15 °C/min) under N_2 atmosphere and kept at this temperature for 55 minutes. After cooling to room temperature, 150 mL EtOH was added as anti-solvent and the suspension was centrifuged for 8 minutes at 1200 RCF. The supernatant was discarded. The precipitate was re-dispersed in 20 mL cyclohexane, 20 mL EtOH was added as anti-solvent and centrifuged for 8 minutes at 1200 RCF. After discarding the supernatant, the precipitate was re-dispersed in 20 mL cyclohexane. The dispersion was centrifuged for 10 minutes at 750 RCF to remove large aggregates and by-products such as NaF. The supernatant was kept as nanocrystal dispersion.

S1.3 Tetrabutylammonium oleate

Tetrabutylammonium oleate (TBA oleate) was synthesised according to a procedure by Khan *et al.*[2] 10 mL of 1.0 M tetrabutylammonium hydroxide in methanol was added with additional (10 mL) methanol to a 100 mL round bottom flask. 2.5 mL OA was added drop-wise while stirring and left for one hour at room temperature. Afterwards, 30 mL of diethyl ether was added. Then, the solvents were evaporated *in vacuo*. The formed residue was redissolved in 20 mL of diethyl ether. The top layer was discarded and the remaining was filtered. The filtrate was dried *in vacuo* until all diethyl ether was evaporated and a yellow ionic liquid was left.

^{1}H-NMR (400 MHz, toluene-d_8) δ (ppm): 5.46 (m, J = 5.5 Hz, 2H), 3.24 (t, J = 8.2 Hz, 8H), 2.44 (t, ^{3}J = 7.7 Hz, 2H), 2.15 – 2.04 (m, 4H), 1.62 – 1.50 (m, 8H), 1.44 (sextet, J = 7.1 Hz, 8H), 1.31-1.25 (m, 20H), 1.04 (t, ^{3}J = 7.2 Hz, 12H), 0.93 (t, ^{3}J = 6.7 Hz, 3H).

S1.4 Yttrium oleate

5 mmol yttrium chloride hexahydrate was dissolved in 12 mL Milli-Q water. 24 mmol sodium oleate, 18 mL ethanol, and 28 mL n-hexane were added. The flask was subjected to three pump-purge cycles of N_2 and vacuum, then left to reflux for 4 hours at 70 °C under N_2 atmosphere. After cooling to room temperature, the organic phase was separated and washed three times with 15 mL of Milli-Q water. Lastly, the remaining hexane was removed using a rotary evaporator to yield the waxy solid yttrium oleate.

S1.5 Undec-10-en-1-ylphosphonic acid

Undec-10-en-1-ylphosphonic acid (UP) was synthesised following a procedure by Knauf *et al.*.[3] 2 mL 11-bromo-1-undecene and 2 mL triethyl phosphite were added to a 25 mL N_2-filled Schlenk flask. This was refluxed at 150 °C for 42 hours. After cooling to room temperature, unreacted triethyl phosphite was removed under vacuum. 10 mL dichloromethane was added, followed by a drop-wise addition of 3.47 mL bromotrimethylsilane. The reaction was left to stir overnight. Subsequently, the excess bromotrimethylsilane was removed *in vacuo*. 10 mL of demineralised water and 10 mL of acetone were added and left to stir for an hour. Solvents were then removed *in vacuo*. 40 mL of pentane was added to the residue, which was then stirred and heated at 50 °C. The hot pentane was slowly transferred into another flask that was put in an ice bath, after which white crystals formed. The pentane was removed *in vacuo*. The product was then recrystallised twice in hot pentane. Lastly, the product was filtered and dried *in vacuo* for 8 hours, resulting in a white powder.

^{1}H-NMR (400 MHz, toluene-d_8) δ (ppm): 6.45 (s, 3H), 5.77 (ddt, J = 13.2, 10.2 , 6.6 Hz, 1H), 5.07 – 4.93 (m, 2H), 1.98 (q, J = 7.2 Hz, 2H), 1.61 (d, J = 9.9 Hz, 4H), 1.31 (m, 2H), 1.23 – 1.08 (m, 14H). ^{31}P NMR (162 MHz, toluene-d_8) δ (ppm): 38.2

S1.6 Oleic acid-*d*1

Oleic acid with the carboxylic acid deuterated was prepared based on Fritzinger *et al.*[4] 200 mg sodium oleate, 2 mL methanol-*d*4, and 1 mL of 1 M DCl in diethyl ether were combined and stirred for an hour, followed by solvent removal with a rotary evaporator. After evaporation, white NaCl powder and liquid oleic acid-*d*1 remain. No acidic proton was visible anymore on the ^{1}H NMR spectrum. Measurements with oleic acid-*d*1 were performed directly after the preparation to prevent prolonged exposure of the deuterated oleic acid to moisture in the air, which would undo the deuteration.

S1.7 NMR measurements

^{1}H NMR measurements were performed at 25 °C unless otherwise specified on a Varian VNMRS400, or a Jeol JNMECZL G 400 MHz spectrometer. Chemical shifts are reported relative to TMS with the residual solvent peak as internal standard. For quantitative NMR measurements p-tolualdehyde was used as internal standard with a relaxation delay of 30 seconds. Titration experiments were performed by adding 10 μL of p-tolualdehyde as an internal standard to a known volume of deuterated toluene containing a known concentration of NCs. An NMR spectrum was then recorded, after which a small amount of OA was added to the NMR tube using a Hamilton microsyringe. The mixture was homogenised by inverting the tube

several times and left to equilibrate for a few minutes before another NMR spectrum was recorded. This process was repeated after successive additions of OA. Solid-state NMR (ssNMR) data were acquired at 9.4 T (400 MHz ^{1}H-frequency) magnetic field using 3.2 mm rotors and a MAS spinning frequency of 10 kHz. ^{13}C-detected ssNMR data were acquired using cross-polarization transfer with a contact time of 2.6 ms and heteronuclear proton decoupling. ^{13}C-chemical shifts were referenced to adamantane.

S1.8 Photoluminescence measurements

Emission spectra were recorded with the sample in a 10x10 mm quartz cuvette using an Edinburgh Instruments Inc FLS920 spectrophotometer with, TMS300 monochromators, and a R928 photomultiplier tube. A 450 W xenon arc lamp was used as excitation source.

S1.9 Transmission electron microscopy

Transmission electron microscopy (TEM) images were taken using a TFS Talos L120C operating at 120 keV. Samples were prepared by drying diluted nanocrystal dispersion on carbon-coated copper grids.

S1.10 X-ray diffraction

Powder X-ray diffraction (XRD) was collected at room temperature using a PANalytical AERIS diffractometer with Cu Kα radiation.

S1.11 Infrared spectroscopy

FT-IR data was recorded on a PerkinElmer SpectrumTwo Infrared Spectrophotometer with an ATR probe.

S1.12 Thermogravimetric analysis

Thermogravimetric analysis (TGA) was performed on a TA Instruments Discovery TGA5500 analyzer with a MKS Cirrus 3 mass spectrometer. Between 1—3 mg of sample was loaded in the analyzer. The samples were first heated to 150 °C in argon for 30 minutes to remove adsorbed water and solvent. Then, they were heated in 20% oxygen in argon with a ramp of 10 °C per min up to 600 °C.

S1.13 Quantifying dV

We determined the volume of solvent trapped with the precipitate during washing (dV) by adding 150 μL ethanol to a 150 μL dispersion of NCs in toluene with the same concentration as used in the washing

experiment shown in Figure 2. This dispersion with total volume 300 μL (also same as Figure 2) is then centrifuged to precipitate the NCs. The tube is decanted and immediately weighed. Then it is left to dry in air, until all solvent is evaporated and weighed again. The weight difference was 16.6 mg, which is equal to 20 μL 50/50 ethanol/toluene solvent. We repeated the experiment with 750 μL dispersion of the same NC concentration and found dV to be 106 μL, approximately the same dV/V ratio. Thus dV depends on the amount of NCs precipitated, while it is independent of V.

S1.14 Model fitting

The amount of OA after one wash as shown in Figure 2 was used as input value for n_i in eq S5, with n_{NC}, V, and dV also input parameters as known from the experimental setup. The equation was iterated 6 times for varying values of K and N_{S}, where the best values were selected as the smallest absolute difference to the data from TGA, with all data point carrying equal weight. Eqs 3 and 4 were fitted to the data in Figure 3c using the method of least squares with all data points carrying equal weight.

S2 NC characterisation

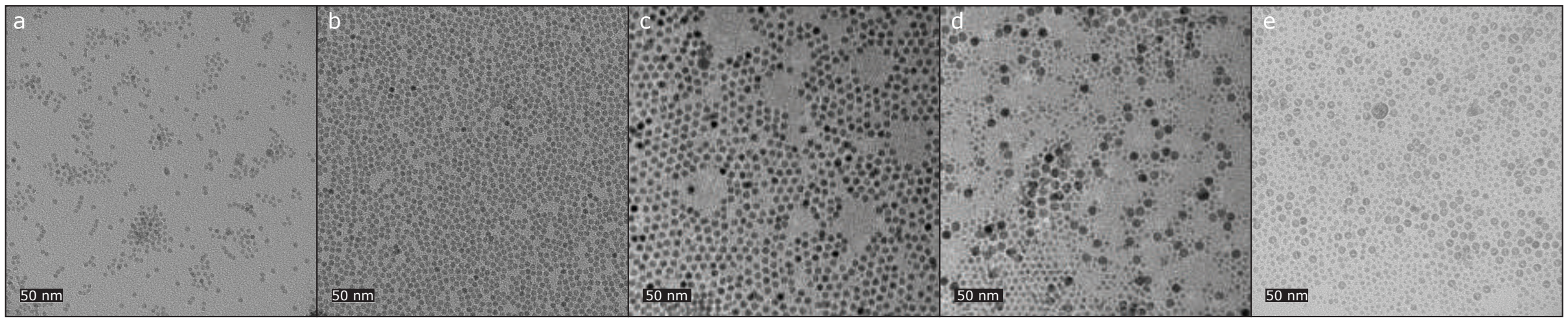

Figure S1: TEM images of (a) $NaYF_4$ NCs used in the washing and TGA experiment and (b)–(e) $NaYF_4$, $NaY_{0.9}Lu_{0.1}F_4$, $NaY_{0.5}Lu_{0.5}F_4$, and $NaLuF_4$ NCs used in the ^{13}C NMR measurements, respectively.

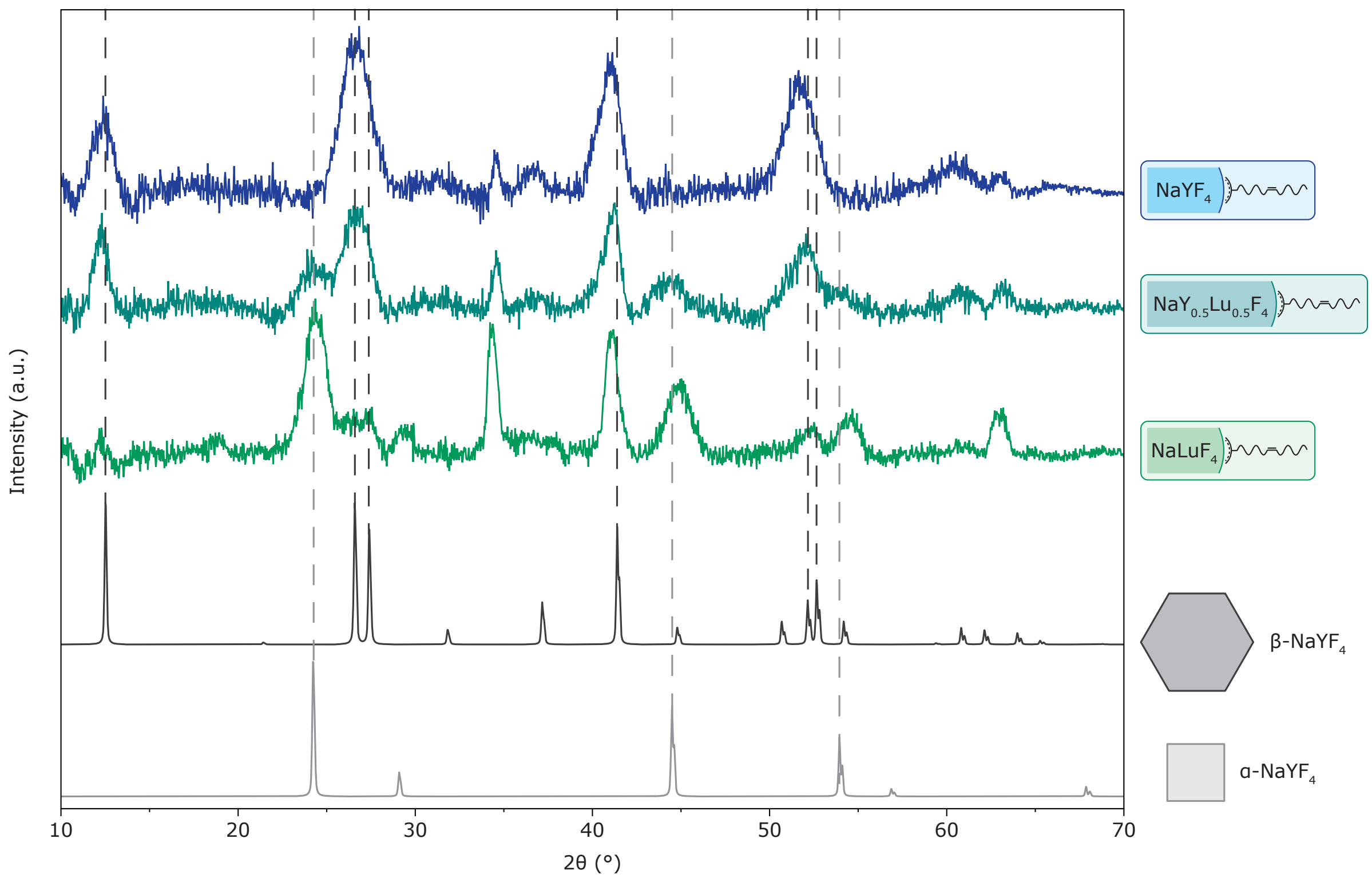

Figure S2: XRD of $NaYF_4$ (Figure S1a), $NaY_{0.5}Lu_{0.5}F_4$ (Figure S1d), and $NaLuF_4$ (Figure S1e) NCs, along with reference peaks for β-$NaYF_4$ (COD ID: 96-151-7673) and α-$NaYF_4$ (COD ID: 96-151-7676).

S3 Full NMR spectra

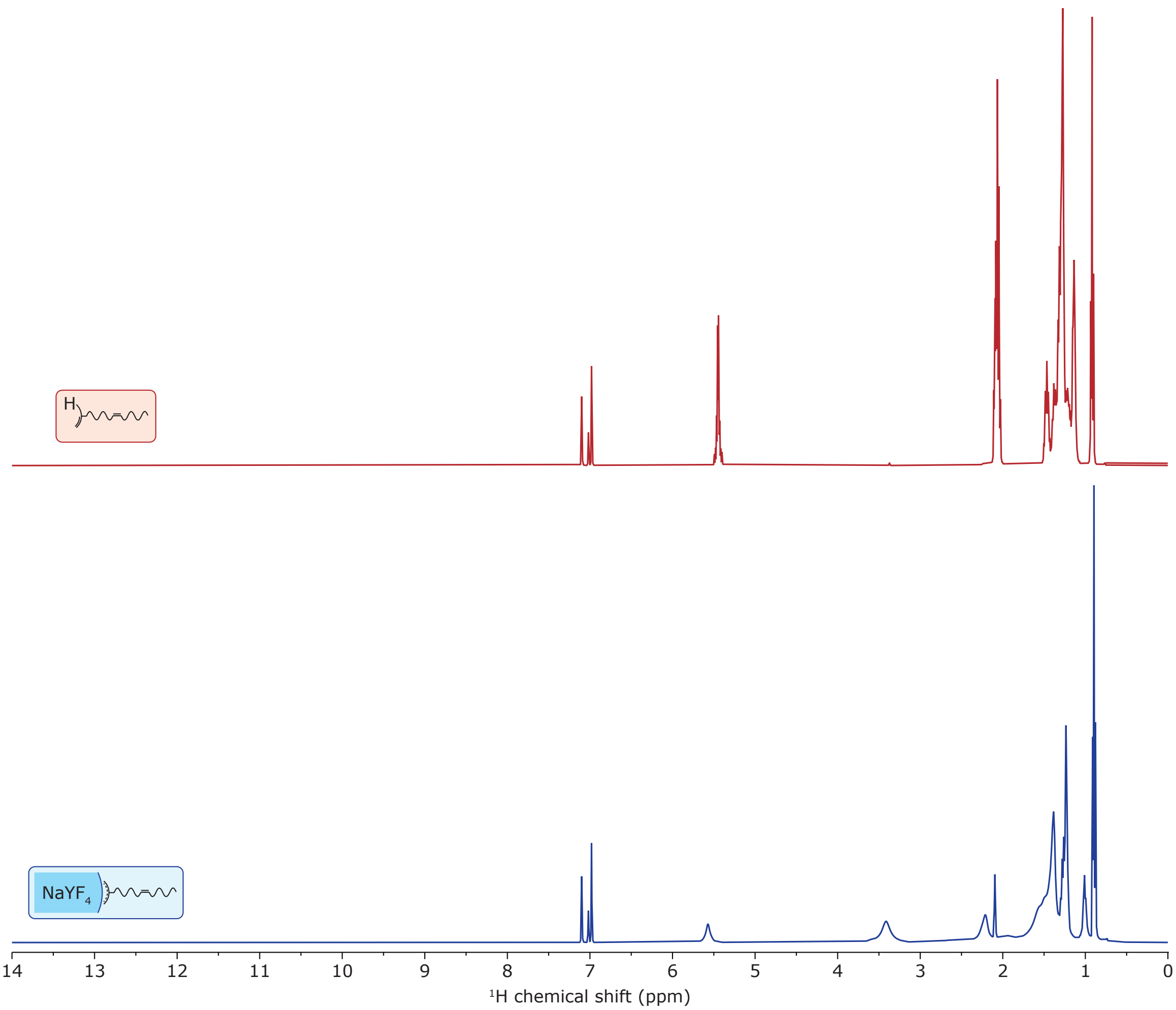

Figure S3: Full ^{1}H NMR spectra of oleic acid (top, red) and oleic acid-capped $NaYF_4$ nanocrystals (bottom, blue) in deuterated toluene with the measured species shown schematically next to the spectra in corresponding colours.

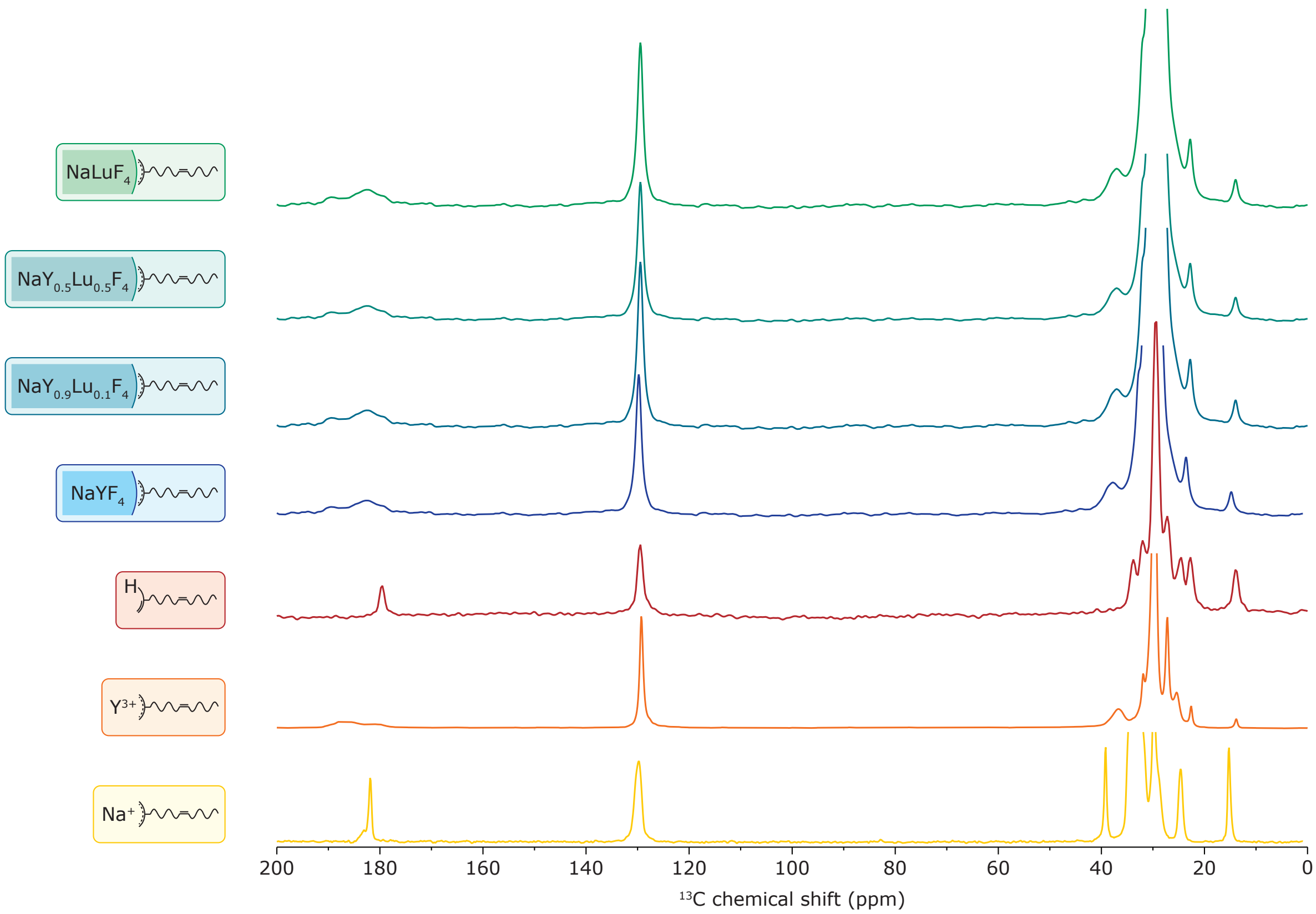

Figure S4: Red: full ^{13}C NMR resonance of oleic acid in deuterated toluene in the region around the carboxyl carbon. The other spectra show the same, but for dried samples in solid-state ^{13}C NMR. Yellow: sodium oleate; orange: yttrium oleate. The blue to green gradient shows dried oleate-capped NCs, where the composition is varied from $NaYF_4$ (blue) to $NaLuF_4$ (green) with $NaY_{0.9}Lu_{0.1}F_4$ and $NaY_{0.5}Lu_{0.5}F_4$ in-between.

S4 Analysis of OA and impurity content during a series of washes

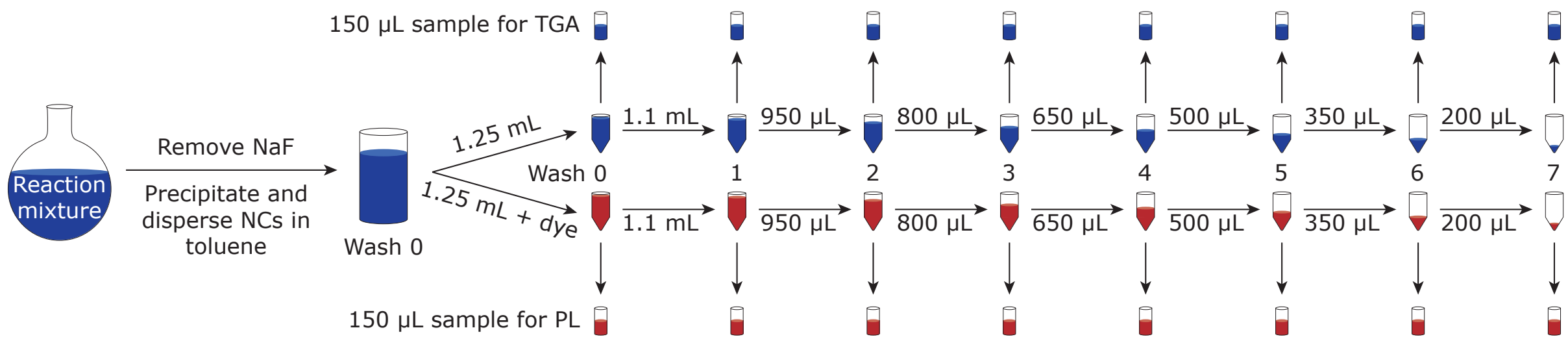

Figure S5: Schematic overview of how the NCs are washed seven consecutive times to study ligand and contaminant content. First, the reaction mixture (100 mL) is centrifuged to remove the NaF by-product. The precipitate is discarded. Ethanol (100 mL) is added to the supernatant, which is then centrifuged to precipitate the NCs. 10 mL toluene is used to re-disperse the NCs. This dispersion is considered wash 0. From this, 1.25 mL is taken to study the effect of washing on ligand density. First, 150 μL is dried to provide a sample for the thermogravimetric analysis of the wash 0 sample. To the remaining 1.1 mL dispersion, 1.1 mL ethanol is added, then centrifuged, followed by re-dispersion in 1.1 mL clean toluene. Again, 150 μL is isolated and dried to serve as a TGA sample of wash 1. The remaining 950 μL is washed and the procedure is repeated for all seven washes. In the modelling we consistently use a washing volume of 300 μL for all samples, as all 150 μL samples would have been dispersed in 150 μL toluene with 150 μL ethanol added before centrifuging if they were a separate dispersion before washing. Using this effective washing volume instead of the total actual washing volume is valid as K is independent of washing volume, as long as the concentration remains equal, since then V, n_{B}, n_{OA}, and n_{S} all scale linearly with V. The procedure for samples studied with photoluminescence is identical, except for the addition of 3.0 mg coumarin 6H. This is added to the 1.25 mL taken from wash 0, but before the 150 μL is isolated.

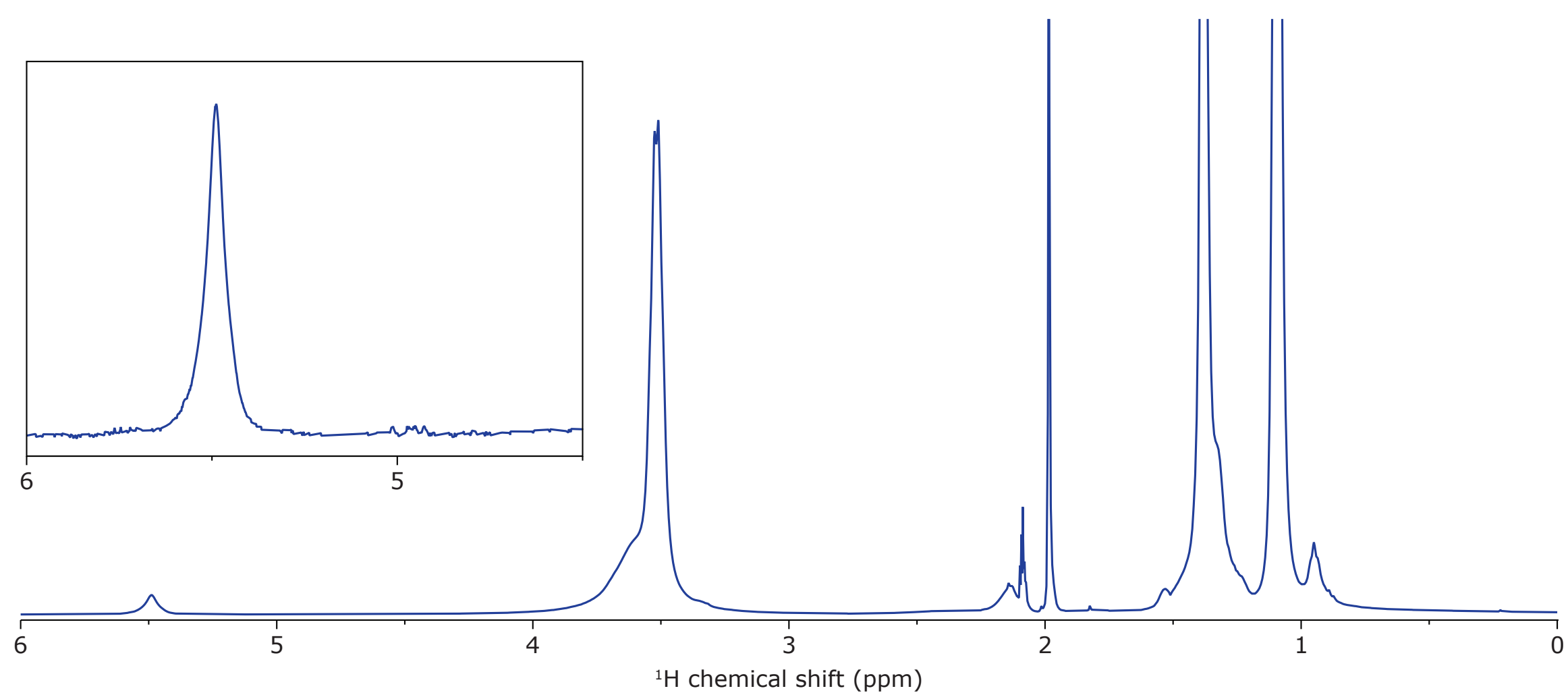

Figure S6: ^{1}H NMR spectrum of $NaYF_4$ NCs in deuterated toluene. These NCs have only been washed once. All resonances can be assigned to OA or toluene, indicating no significant contaminants remain. ODE would be a major concern and while its alkane signals would be hard to distinguish from OA, no signals beside the two OA protons are seen in the alkene region between 6 and 4.5 ppm (enlarged).

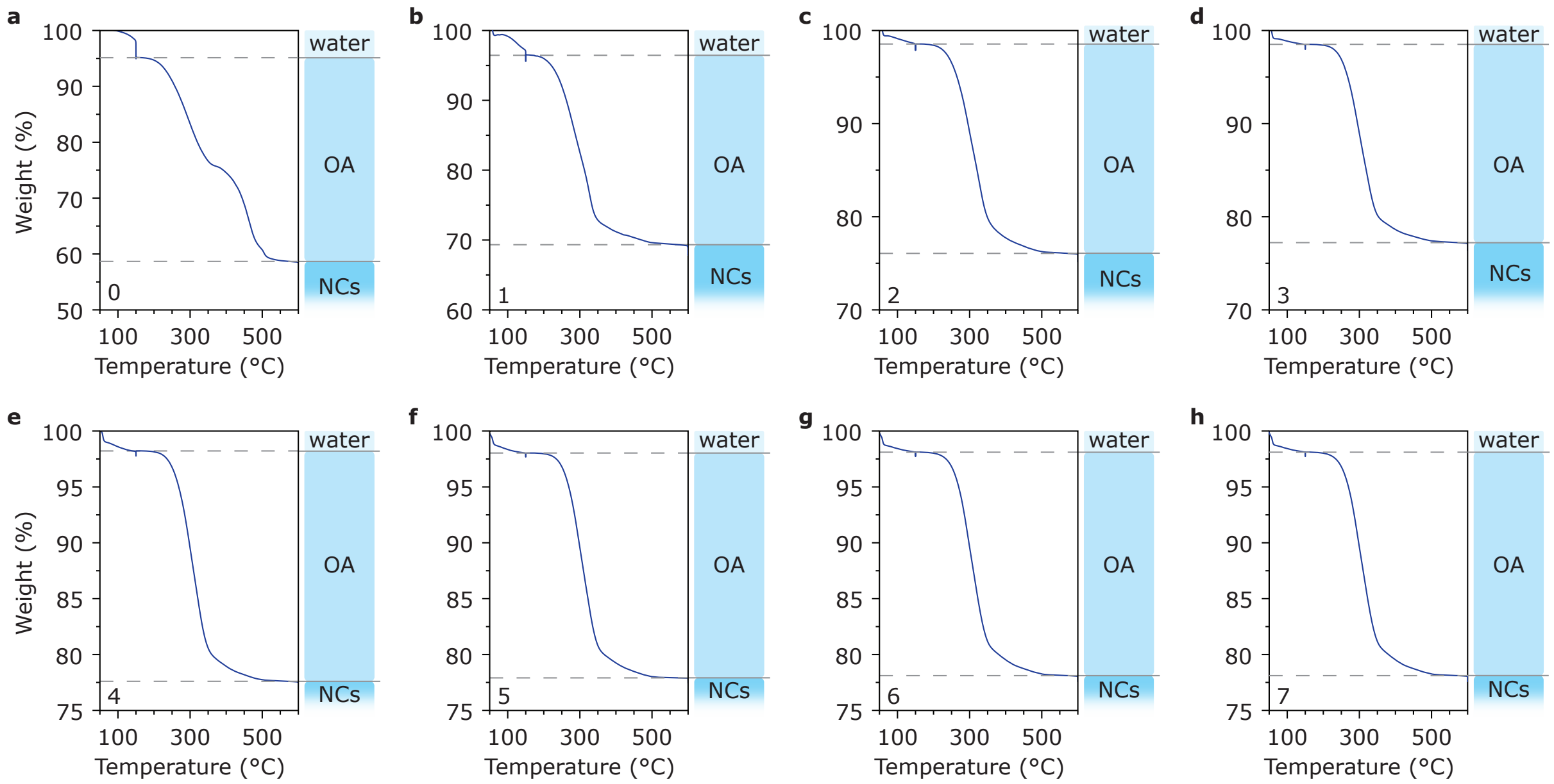

Figure S7: Thermogravimetric analysis (TGA) curves before (a) and after each of seven washes (b–h). The small drop below 200 °C is attributed to adsorbed water and solvent.[5] Between 200 °C and 500 °C OA is combusted, reducing the weight significantly. Beyond 500 °C the weight is stable and only the NCs remain. The curve before any washing has a significant shoulder around 400 °C from some additional species, likely ODE. This makes it impossible to accurately determine OA content in this sample. Therefore, data is only analysed from the first wash (b) onwards, which showed no significant contamination (Figure S6), corresponding to the samples labelled wash 1–7 in Figure S5. Only 1–3 mg of the approximately 14 mg dried NCs from the isolated 150 μL sample is used for TGA. To accurately calculate the total OA content of the whole sample from the OA content of the TGA sample, we use the OA weight loss relative to the NC weight. Knowing the amount of NCs in dispersion and assuming it stays constant with washing (which is a reasonable assumption as the dried aliquot weight does not drop significantly across the washing series), the total OA can be determined from its relative amount to NCs in the TGA sample.

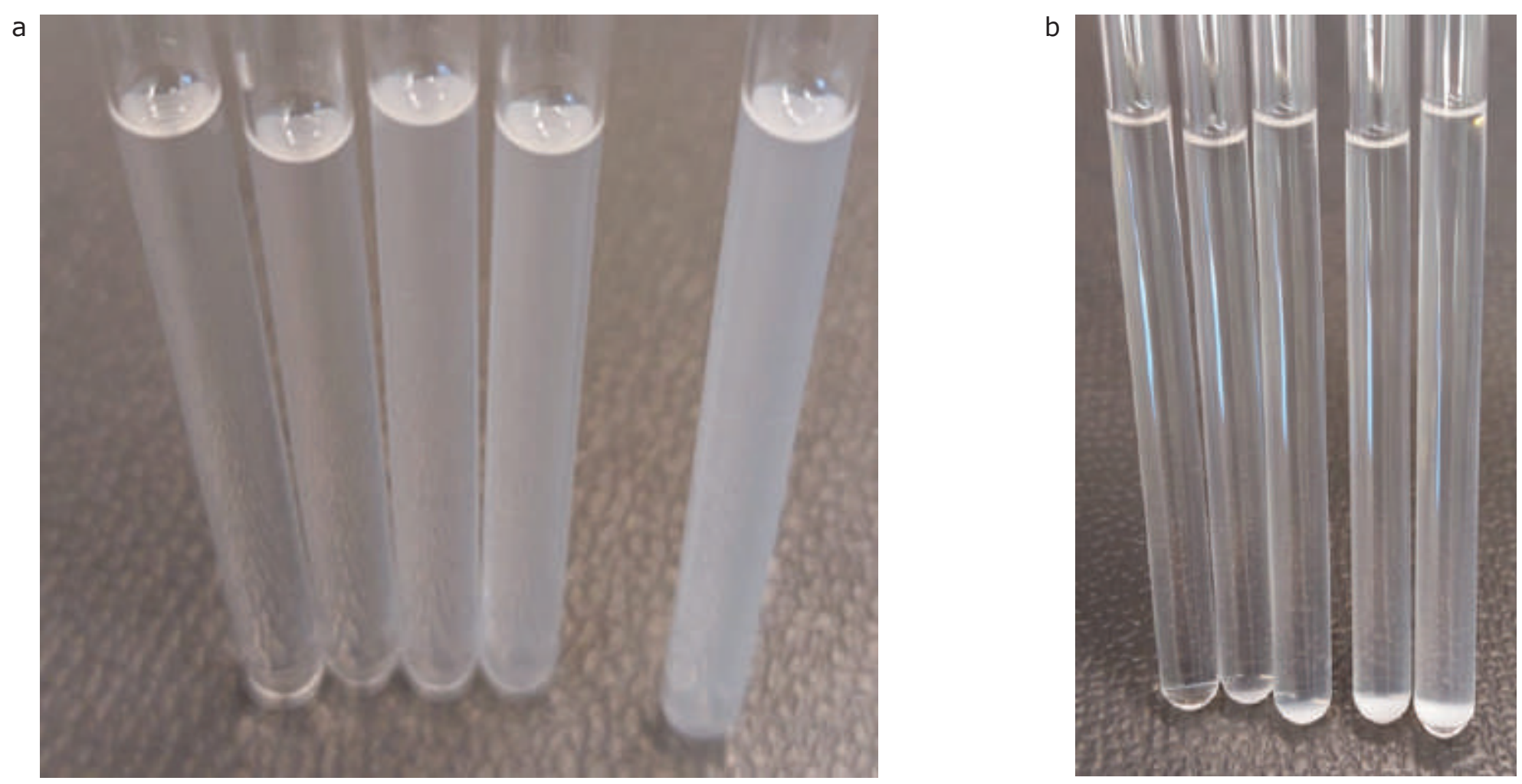

Figure S8: NCs washed 1–5 times (from left to right) in deuterated toluene. (a) Just after shaking the tubes. The dispersion grows progressively more cloudy with number of washes, indicating some aggregation and colloidal instability. (b) After leaving these dispersions approximately 30 minutes, significant precipitation of NCs can be seen when they have been washed multiple times. Using eq 1 and fit values found, we find that 99.4%, 96.2%, 93.4%, 91.3%, and 89.5% of the binding sites are filled with OA after 1 to 5 washes, respectively. Thus, a small reduction in ligand coverage seems to have a significant effect on the colloidal stability of NCs.

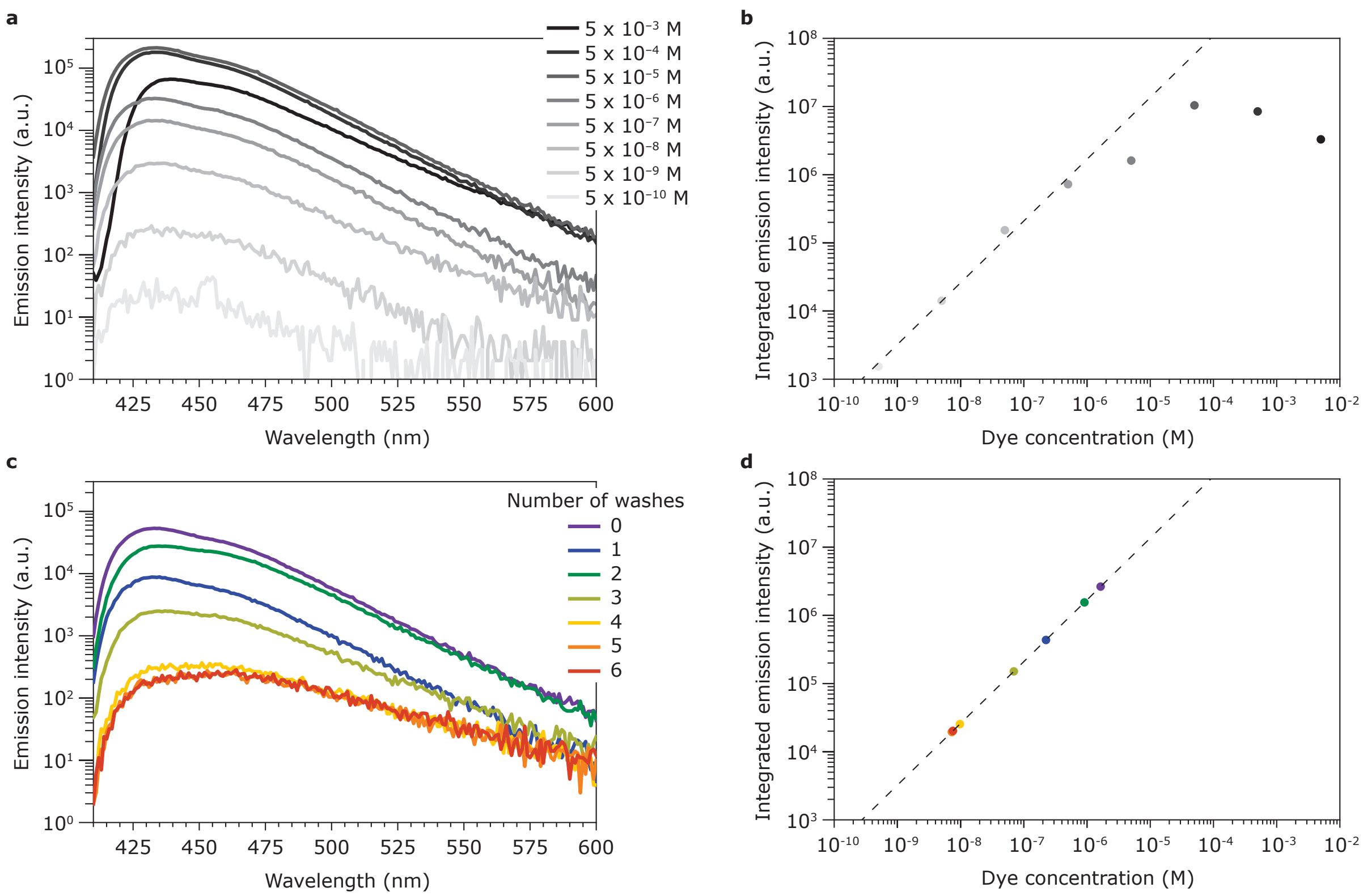

Figure S9: (a) Emission spectra of different concentrations of coumarin 6H in toluene upon 400 nm excitation. (b) Integrated intensities of spectra (a) over the whole range shown (410–600 nm), plotted against concentration of the dye. At high concentration, the integrated intensity deviates from the expected linear trend as seen at low concentration, so only the lowest 4 concentrations are used for the calibration line (dashed). (c) Same as (a) but of the samples as prepared according to Figure S5. The 150 μL samples taken from washing were diluted ten times to provide sufficient volume for the measurements. The samples after 0 and 1 washes were diluted an additional 1000 and 500 times, respectively, so that their photoluminescent intensity fall within the linear response. (d) The integrated intensities of the spectra shown in (c), plotted on the calibration line from (b). Dividing the concentrations found from this calibration line (while accounting for the dilution in wash 0 and 1) by the concentration before any washing (wash 0) yields the data points in Figure 2d.

S5 Variable-temperature ^{1}H NMR on NCs with excess OA

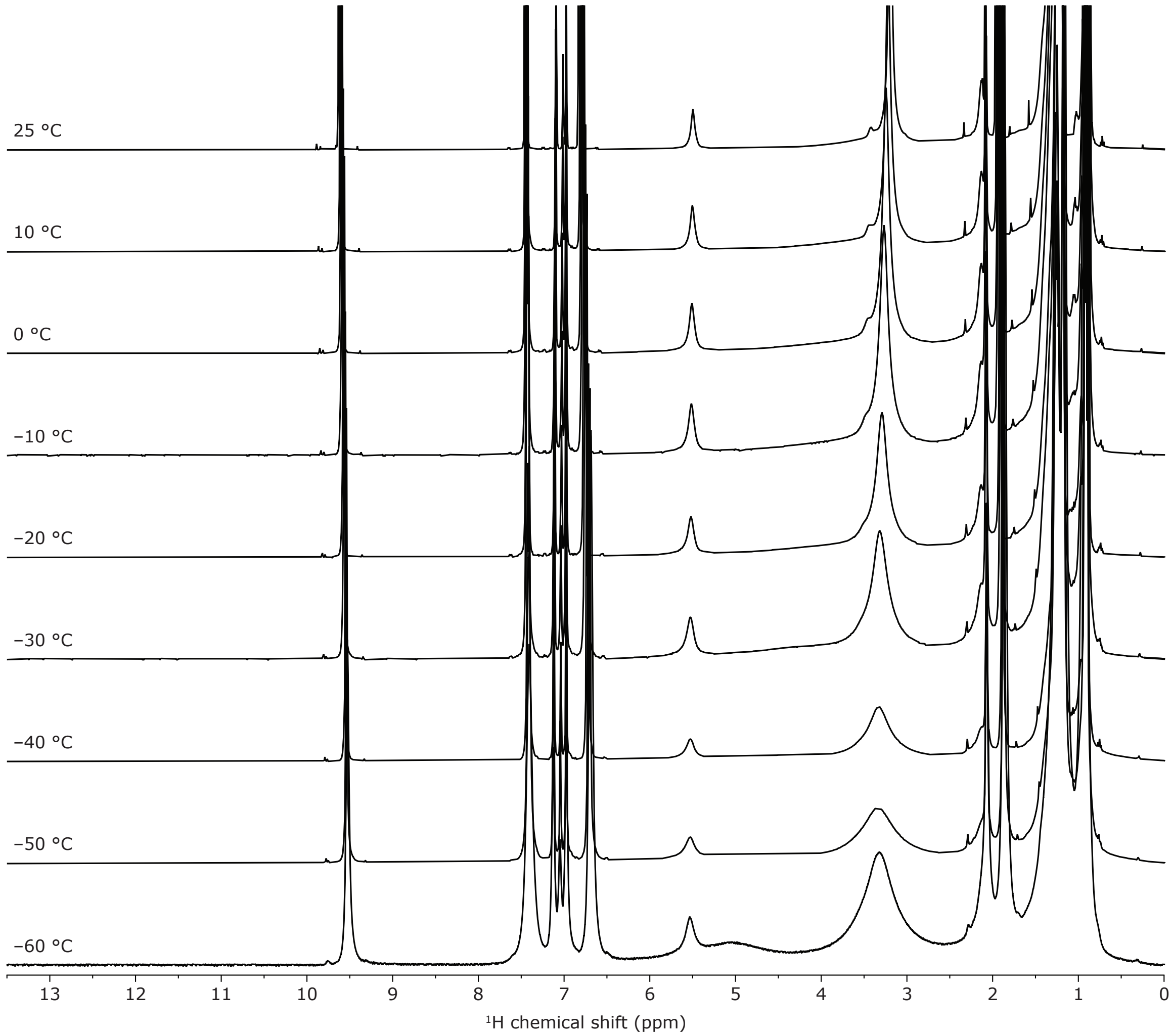

Figure S10: ^{1}H NMR spectra of OA-capped NCs with 10 μL p-tolualdehyde (internal standard) and 5 μL free OA added ranging from 25 °C (top) to –60 °C (bottom). Across the whole temperature range, a single alkene peak is seen around 5.6 ppm, indicating fast (exceeding 115 s^{-1}) exchange between bound and free OA at all temperatures.

S6 Binding–unbinding equilibrium of OA

Eq S1 describes the equilibrium between OA free in solution (n_F) and OA bound to NCs (n_B) in units of mol:

$$\frac{n_\mathrm{F}}{V} + \frac{n_\mathrm{V}}{V} \overset{K}{\rightleftarrows} \frac{n_\mathrm{B}}{V} \tag{S1}$$

with n_V the number of vacant binding sites on all NC surfaces in mol and V the volume of the OA-capped NC dispersion. K is thus the equilibrium constant defined as

$$K = \frac{n_\mathrm{B} V}{n_\mathrm{F} n_\mathrm{V}} \tag{S2}$$

for the binding/unbinding in equilibrium. By realising that the amount of free OA equals the total amount of OA n_OA minus the amount of bound OA and the number of vacancies equals the total number of binding sites n_S again minus the bound OA concentration, we rewrite eq S2 as

$$K = \frac{n_\mathrm{B} V}{(n_\mathrm{OA} - n_\mathrm{B})(n_\mathrm{S} - n_\mathrm{B})} \tag{S3}$$

With $n_\mathrm{S} = N_\mathrm{S} n_\mathrm{NC}$ we arrive at eq 1 from the main text, with n_S the number of binding sites per NC and n_NC the amount of NCs in dispersion (unit: mol). Solving for n_B gives

$$n_\mathrm{B} = \frac{1}{2}\left(\frac{V}{K} + N_\mathrm{S} n_\mathrm{NC} + n_\mathrm{OA} - \sqrt{\left(\frac{V}{K} + N_\mathrm{S} n_\mathrm{NC} + n_\mathrm{OA}\right)^2 - 4 N_\mathrm{S} n_\mathrm{NC} n_\mathrm{OA}}\right) \tag{S4}$$

Mathematically, the positive root is also a valid solution. However, as the concentration of bound OA can never be larger than the total OA concentration and all variables have positive values, only the negative root is physically meaningful.[6]

This equation describes the amount of OA that is bound in a dispersion, such as described in Figure 2a, stage II. In stage V, the free OA has been decanted except for the molecules trapped in volume dV. The amount of OA taken over to washing step $i+1$ are the bound molecules in stage II of washing step i plus the molecules trapped in volume dV of washing step i:

$$n_{i+1} = n_{\mathrm{B}} + \frac{\mathrm{d}V}{V} n_{\mathrm{F}} = n_{\mathrm{B}} + (n_i - n_{\mathrm{B}}) \frac{\mathrm{d}V}{V} \tag{S5}$$

where $\mathrm{d}V/V$ is the fraction of volume trapped and n_i is the amount of OA before the wash in stage II, equivalent to n_{OA} in eq S4. Therefore, eqs S4 and S5 can be combined to give

$$n_{i+1} = \frac{\mathrm{d}V}{V} n_i + \frac{1}{2}\left(1 - \frac{\mathrm{d}V}{V}\right)\left(\frac{V}{K} + N_{\mathrm{S}} n_{\mathrm{NC}} + n_i - \sqrt{\left(\frac{V}{K} + N_{\mathrm{S}} n_{\mathrm{NC}} + n_i\right)^2 - 4 N_{\mathrm{S}} n_{\mathrm{NC}} n_i}\right) \tag{S6}$$

The values of N_{S} and K are found by fitting the iteration of S6 to the data points in Figure 2b.

S7 Two ligands in equilibrium

Eq S2 can be written more generally for any ligand L:

$$K_{\mathrm{L}} = \frac{n_{\mathrm{L,B}} V}{n_{\mathrm{L,F}} n_{\mathrm{V}}} \tag{S7}$$

The ratio between K_{L} of any titrating ligand L and K_{OA} can then be written as

$$\frac{K_{\mathrm{L}}}{K_{\mathrm{OA}}} = \frac{n_{\mathrm{L,B}} n_{\mathrm{OA,F}}}{n_{\mathrm{L,F}} n_{\mathrm{OA,B}}} \tag{S8}$$

where we added explicit OA labels to the bound and free OA amounts for clarity. As the two ligands compete for the same vacancies on the NC, this amount n_{V} gets divided out, along with volume V. Realising that the bound titrating ligand is equal to the number of binding sites (n_{S}) minus the bound OA and using $n_{\mathrm{F}} = n_{\mathrm{L}} - n_{\mathrm{B}}$ for L and OA gives

$$\frac{K_{\mathrm{L}}}{K_{\mathrm{OA}}} = \frac{(n_{\mathrm{S}} - n_{\mathrm{OA,B}})(n_{\mathrm{OA}} - n_{\mathrm{OA,B}})}{(n_{\mathrm{L}} - n_{\mathrm{S}} + n_{\mathrm{OA,B}}) n_{\mathrm{OA,B}}} \tag{S9}$$

Solving for $n_{\mathrm{OA,B}}$:

$$n_{\mathrm{OA,B}} = \frac{\frac{K_{\mathrm{L}}}{K_{\mathrm{OA}}} n_{\mathrm{S}} - \frac{K_{\mathrm{L}}}{K_{\mathrm{OA}}} n_{\mathrm{L}} - n_{\mathrm{S}} - n_{\mathrm{OA}} + \sqrt{\left(-\frac{K_{\mathrm{L}}}{K_{\mathrm{OA}}} n_{\mathrm{S}} + \frac{K_{\mathrm{L}}}{K_{\mathrm{OA}}} n_{\mathrm{L}} + n_{\mathrm{S}} + n_{\mathrm{OA}}\right)^2 + 4\left(\frac{K_{\mathrm{L}}}{K_{\mathrm{OA}}} - 1\right) n_{\mathrm{S}} n_{\mathrm{OA}}}}{2\left(\frac{K_{\mathrm{L}}}{K_{\mathrm{OA}}} - 1\right)} \tag{S10}$$

Here, $n_{\mathrm{L}}, n_{\mathrm{S}}$, and n_{OA} are known from experimental procedure or previous fits, so the only unknown parameter is $K_{\mathrm{L}}/K_{\mathrm{OA}}$, which is found by fitting to the data points in Figure 4.

The experiment where we titrate additional OA is a special case where $K_{\mathrm{L}} = K_{\mathrm{OA}}$. In this case, the coefficient of the quadratic term in eq S9 becomes zero. Consequently, eq S10, which was obtained using the quadratic formula, is no longer applicable. Instead, eq S9 reduces to a linear equation, yielding the simpler solution

$$n_{\mathrm{OA,B}} = \frac{n_{\mathrm{S}} n_{\mathrm{OA}}}{n_{\mathrm{L}} + n_{\mathrm{OA}}} \tag{S11}$$

The fraction of OA that remains bound can then be written as

$$f_{\mathrm{OA,B}} = \frac{n_{\mathrm{OA,B}}}{n_{\mathrm{OA}}} = \frac{n_{\mathrm{OA,init}}}{n_{\mathrm{OA,init}} + n_{\mathrm{OA,added}}} \tag{S12}$$

Here, $f_{\mathrm{OA,B}}$ is the fraction of OA that remains bound, as plotted in Figure 4c. In obtaining this expression, we assumed that initially all binding sites are occupied by OA and that all initially present OA ($n_{\mathrm{OA,init}}$) is therefore bound. Since the titrating ligand L is added to the system, $n_{\mathrm{L}} = n_{\mathrm{OA,added}}$.

References

(1) Homann, C.; Krukewitt, L.; Frenzel, F.; Grauel, B.; Würth, C.; Resch-Genger, U.; Haase, M. NaYF4:Yb,Er/NaYF4 Core/Shell Nanocrystals with High Upconversion Luminescence Quantum Yield. *Angewandte Chemie* **2018**, *130*, 8901–8905.

(2) Khan, Y.; Hwang, S.; Braveenth, R.; Jung, Y. H.; Walker, B.; Kwon, J. H. Synthesis of fluorescent organic nano-dots and their application as efficient color conversion layers. *Nat. Commun.* **2022**, *13*.

(3) Knauf, R. R.; Lennox, J. C.; Dempsey, J. L. Quantifying ligand exchange reactions at CdSe nanocrystal surfaces. *Chem. Mater.* **2016**, *28*, 4762–4770.

(4) Fritzinger, B.; Capek, R. K.; Lambert, K.; Martins, J. C.; Hens, Z. Utilizing self-exchange to address the binding of carboxylic acid ligands to CdSe quantum dots. *J. Am. Chem. Soc.* **2010**, *132*, 10195–10201.

(5) Li, Z.; Miao, H.; Fu, Y.; Liu, Y.; Zhang, R.; Tang, B. Fabrication of NaYF4:Yb,Er Nanoprobes for Cell Imaging Directly by Using the Method of Hydrion Rivalry Aided by Ultrasonic. *Nanoscale Res. Lett.* **2016**, *11*.

(6) Donakowski, M. D.; Godbe, J. M.; Sknepnek, R.; Knowles, K. E.; de la Cruz, M. O.; Weiss, E. A. A Quantitative Description of the Binding Equilibria of para-Substituted Aniline Ligands and CdSe Quantum Dots. *J. Phys. Chem. C* **2010**, *114*, 22526–22534.
